# Supplementary material for: Effect of Slow-Release Urea on Yield and Quality of Euryale ferox
Source: Int J Mol Sci. 2024 Oct 31;25(21):11737. doi: 10.3390/ijms252111737 (PMC11546189; doi:10.3390/ijms252111737)
Supplement: Supplementary file 1 [file ijms-25-11737-s001.zip › ijms-3243852- SF.pdf]

International Journal of
Molecular Sciences

MDPI

Article

Effect of Slow-Release Urea on Yield and Quality of *Euryale ferox*

Peng Wu [1,†], Tian-Yu Wang [1,†], Yu-Hao Wang [1], Ai-Lian Liu [1], Shu-Ping Zhao [1], Kai Feng [1] and Liang-Jun Li [1,2,*]

[1] School of Horticulture and Landscape Architecture, Yangzhou University, Wenhui East Road No. 48, Yangzhou 225009, China; wupeng@yzu.edu.cn (P.W.); 18852712804@163.com (T.-Y.W.); wangyuhaoyzu@163.com (Y.-H.W.); liuailianyz@163.com (A.-L.L.); zhaoshuping@yzu.edu.cn (S.-P.Z.); fengkai@yzu.edu.cn (K.F.)

[2] Joint International Research Laboratory of Agriculture and Agri-Product Safety of Ministry of Education of China, Yangzhou University, Yangzhou 225009, China

* Correspondence: ljli@yzu.edu.cn

† These authors contributed equally to this work.

Supplemenatry Figures

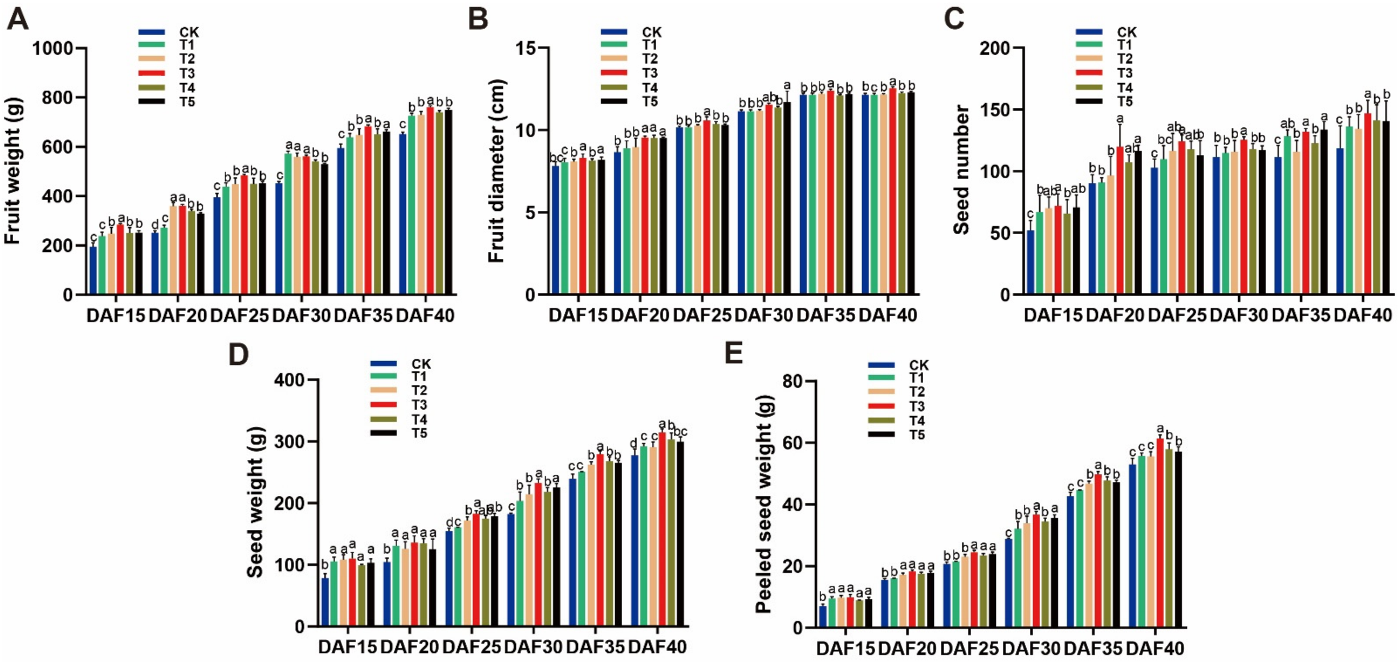

Figure S1. Yield determination under different SRU quantity of application(CK and T1-T5). A, Yield per plant. B, Total yield. Different letters indicate significantly different values (p<0.05).

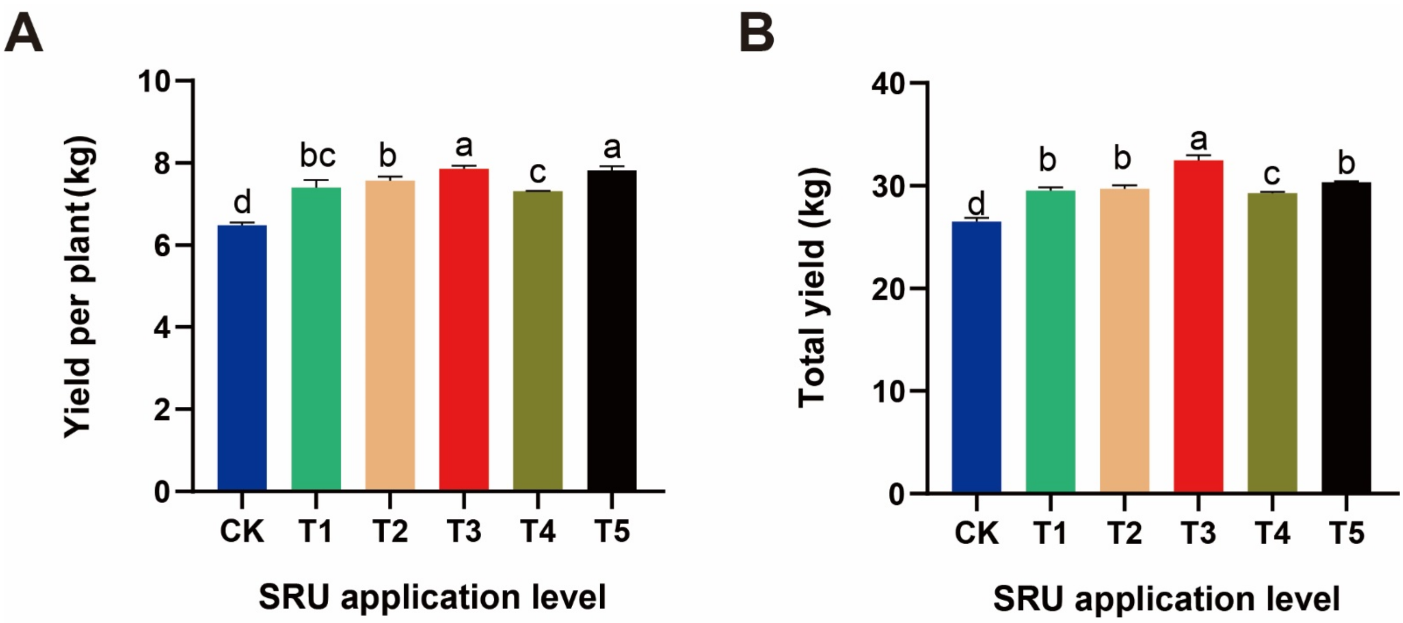

Figure S2. Enzyme activity under different SRU quantity of application(CK and T3). A, SBE. B, SSS. Different letters indicate significantly different values (p<0.05).

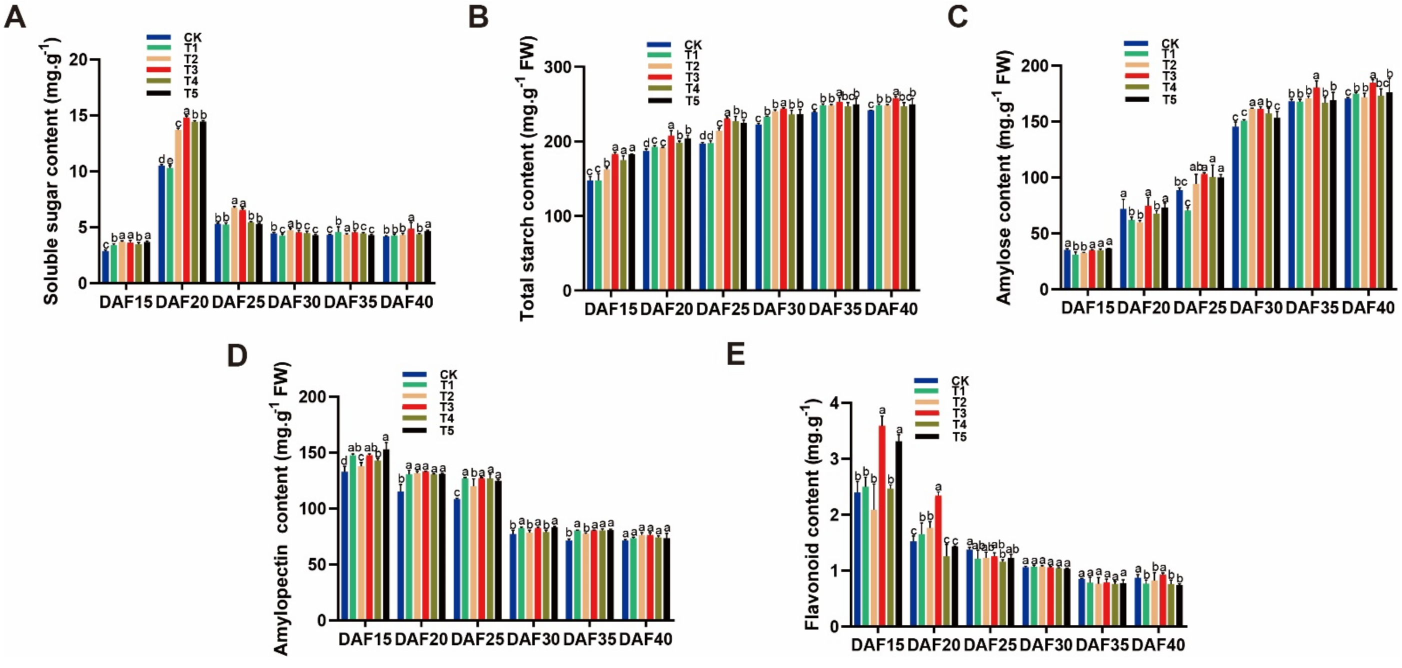

Figure S3. Yield determination under different period of application of SRU(CK and S1-S4). A, Yield per plant. B, Total yield. Different letters indicate significantly different values (p<0.05).

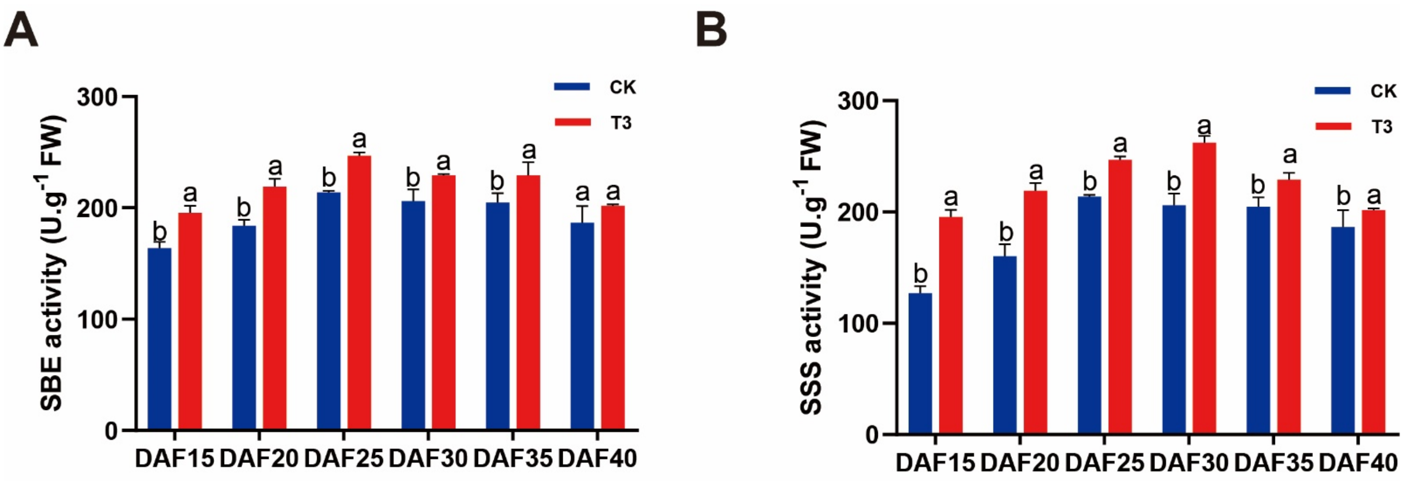

Figure S4. Enzyme activity under different period of application of SRU(CK and S2). A, SSS. B, SBE. C, GBSS. Different letters indicate significantly different values (p<0.05).

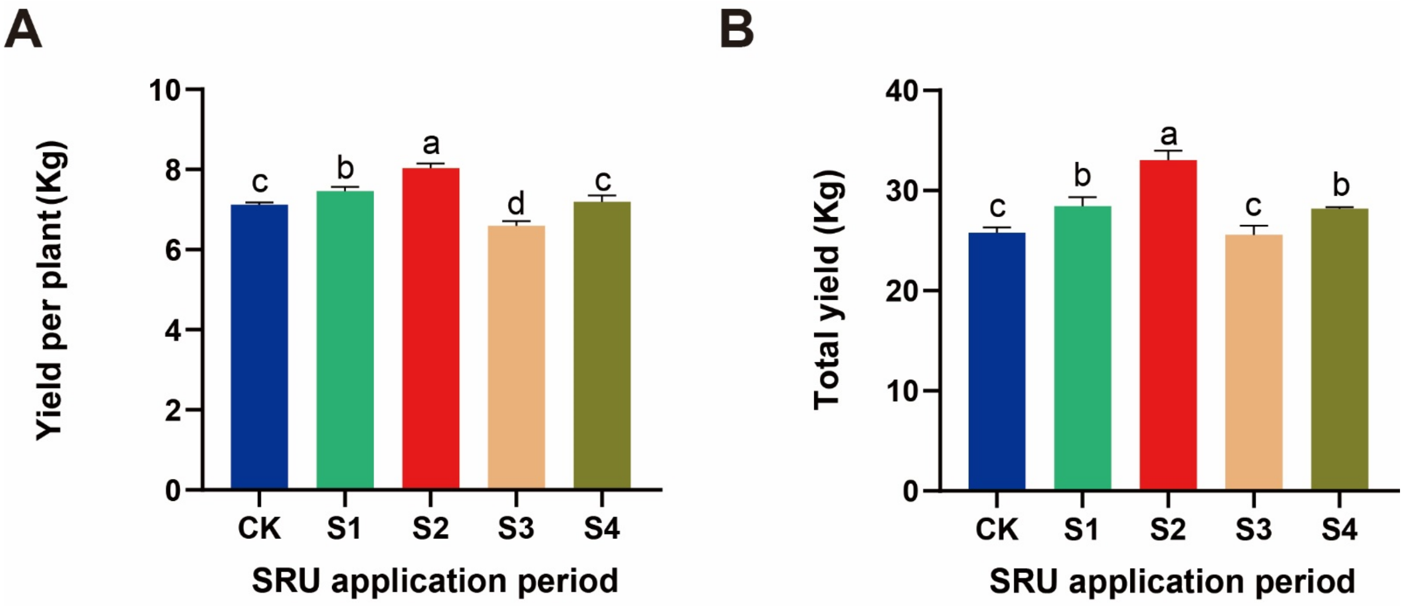

Figure S5. Physiological data of *E. ferox* under different SRU quantity of application(CK and T1-T5). A, Fruit weight. B, Fruit diameter. C, Seed number. D, Seed weight. E, Peeled seed weight. Different letters indicate significantly different values (p<0.05).

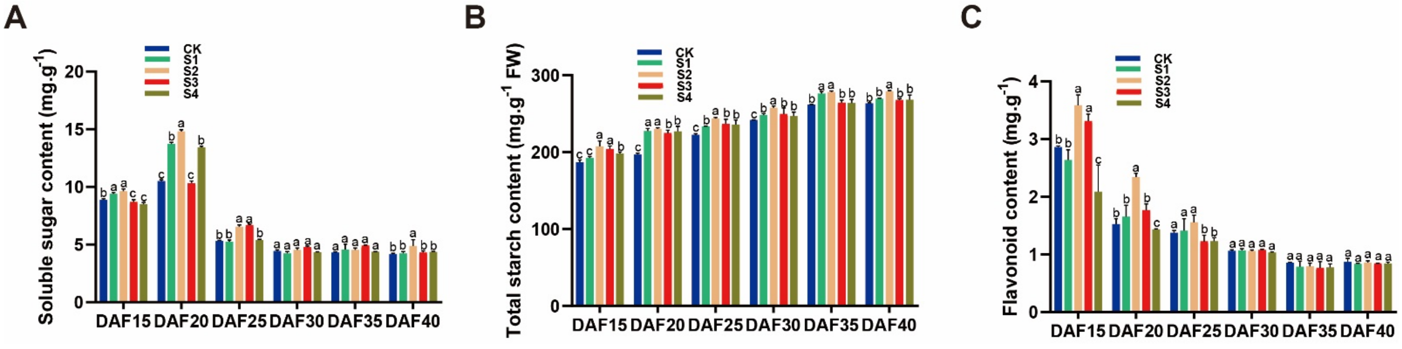

Figure S6. Content of each indicator under different SRU quantity of application(CK and T1-T5). A, Soluble sugars. B, Total starch. C, Amylose. D, Amylopectin. E, Flavonoids. Different letters indicate significantly different values (p<0.05).

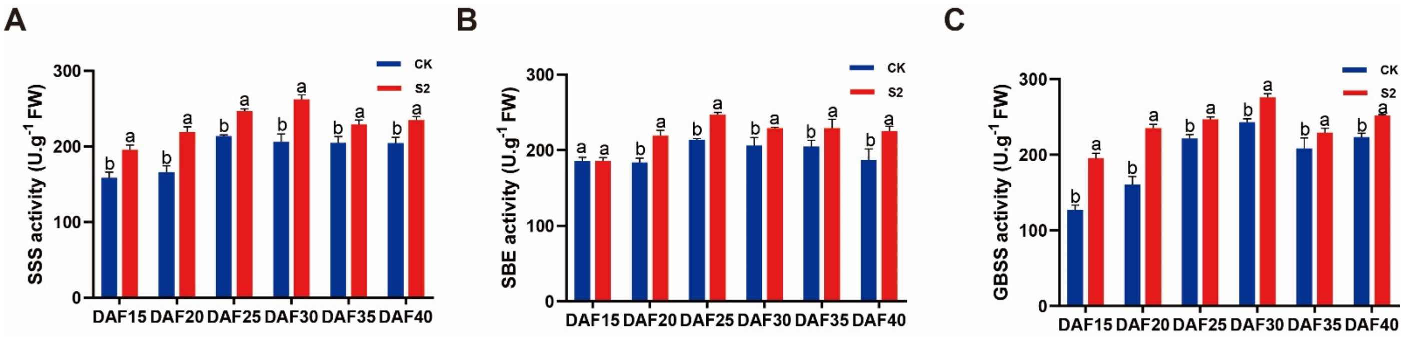

Figure S7. Physiological data of E. ferox under different period of application of SRU(CK and S1-S4). A, Fruit weight. B, Fruit diameter. C, Seed number. D, Seed weight. E, Peeled seed weight. Different letters indicate significantly different values (p<0.05).

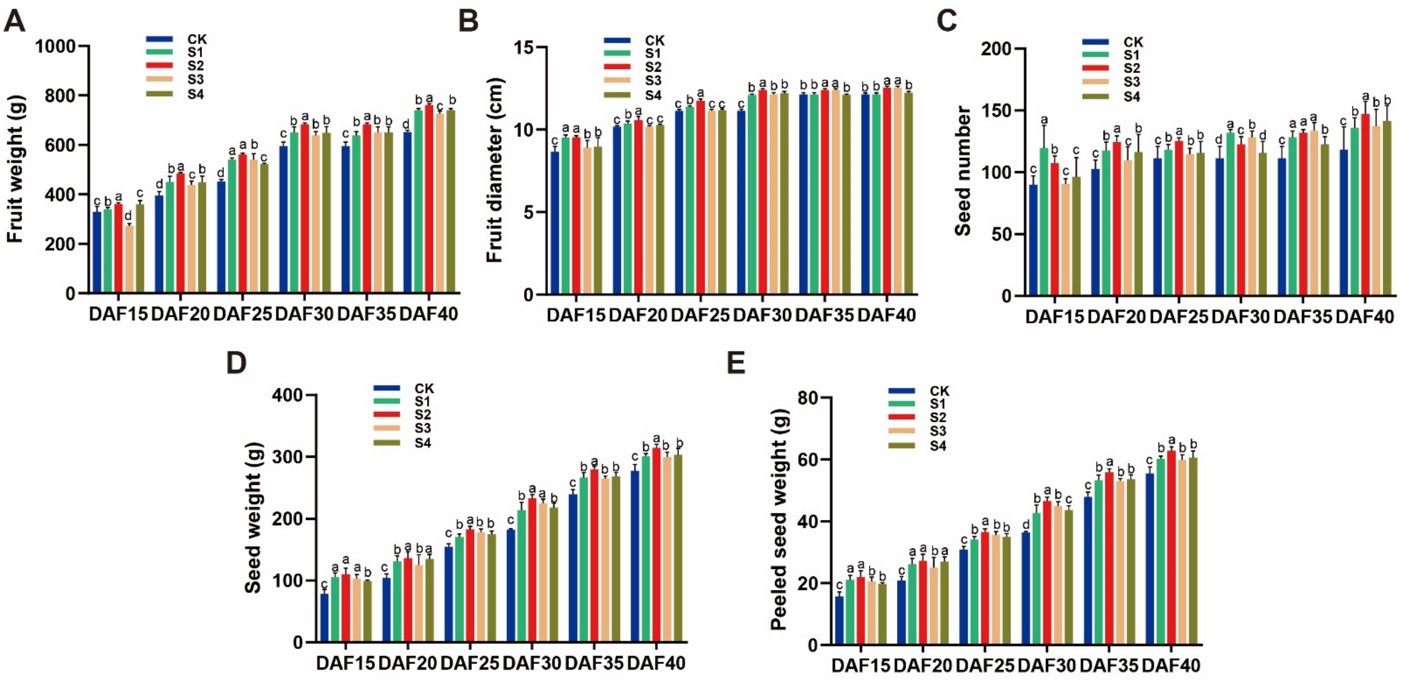

Figure S8. Content of each indicator under different period of application of SRU(CK and S1-S4). A, Soluble sugars. B, Total starch. C, Flavonoids. Different letters indicate significantly different values (p<0.05).

Disclaimer/Publisher's Note: The statements, opinions and data contained in all publications are solely those of the individual author(s) and contributor(s) and not of MDPI and/or the editor(s). MDPI and/or the editor(s) disclaim responsibility for any injury to people or property resulting from any ideas, methods, instructions or products referred to in the content.
